# In-Depth Analysis of *Olea europaea* L. Leaf Extract: Alleviating Pulmonary Histological Disturbances, Pro-Inflammatory Responses, and Oxidative Stress from Isolated or Combined Exposure to Inhaled Toluene and Noise in Rats

**DOI:** 10.3390/biology13110896

**Published:** 2024-11-03

**Authors:** Takoua Ben Attia, Sana Bahri, Sonia Ben Younes, Afef Nahdi, Ridha Ben Ali, Linda Bel Haj Kacem, Michèle Véronique El May, Eduardo Alberto López-Maldonado, Abada Mhamdi

**Affiliations:** 1 Laboratory of Research “LR 23/ES/10”: Population Pathology, Environmental Aggressors, Alternative Therapy, Faculty of Medicine of Tunis, University of Tunis El Manar, Tunis 1068, Tunisia; benyounessonia@gmail.com (S.B.Y.); afef.nahdi@gmail.com (A.N.); ridhabenali9@gmail.com (R.B.A.); elmay_michele@yahoo.fr (M.V.E.M.); abada.mhamdi@fmt.utm.tn (A.M.); 2 Laboratory of Physiology, Faculty of Medicine of Tunis, University of Tunis El Manar, Tunis 1068, Tunisia; bahrisana88@gmail.com; 3 Laboratory of Physiopathology, Food and Biomolecules (LR-17-ES-03), Technology Center of Sidi Thabet, University of Manouba, Tunis 2010, Tunisia; 4 Laboratory of Quality Control, Herbes De Tunisie, Company AYACHI-Group, Mansoura, Siliana 6131, Tunisia; 5 Faculty of Sciences of Gafsa, University of Gafsa, Campus Sidi Ahmed Zarroug, Gafsa 2112, Tunisia; 6 Research Unit n° 17ES15, Department of Pathology, Charles Nicolle Hospital, University of Tunis El Manar, Tunis 1068, Tunisia; linda.belhadjkacem@fmt.utm.tn; 7 Faculty of Chemical Sciences and Engineering, Autonomous University of Baja California, Tijuana CP 22390, BC, Mexico

**Keywords:** *Olea europaea* L. leaf extract, noise, toluene, lung tissue, rat, antioxidant activity

## Abstract

The primary objective of this study was to investigate the pulmonary damage resulting from isolated or combined exposure to inhaled toluene (300 ppm) and noise 85 dB (A), with a focus on evaluating the potential protective effects of *Olea europaea* L. leaf extract (OLE). Forty-eight male *Wistar* rats were divided into eight groups: control (C), OLE treatment (O), noise exposure (N), noise exposure with OLE treatment (N+OLE), toluene exposure (T), toluene exposure with OLE treatment (T + OLE), co-exposure to toluene and noise (NT), and co-exposure with OLE treatment (NT + OLE). OLE (40 mg/kg/day) was administered daily for six weeks via oral gavage. Exposure to toluene and noise resulted in significant disruption of the pulmonary tissue structure, accompanied by oxidative stress, as evidenced by increased lipid peroxidation, diminished catalase and superoxide dismutase activities, and elevated pro-inflammatory cytokines IL6, IL-β, and TNF-α. Notably, the administration of OLE effectively mitigated oxidative stress and inflammation and preserved pulmonary histology. In conclusion, exposure to toluene and its combination with noise significantly elevated oxidative stress, inflammatory responses, and histological disruptions in the lung tissue. In contrast, noise exposure alone is characterized by minimal effects, although it is still associated with an inflammatory response. Notably, *Olea europaea* L. leaf extract (OLE) exhibits a substantial protective role, effectively mitigating the adverse effects of combined exposure and highlighting its potential as a therapeutic agent for lung health.

## Simple Summary

This study investigated the impact of toluene and noise exposure on pulmonary damage, focusing on the potential protective effects of *Olea europaea* leaf extract (OLE). Forty-eight male *Wistar* rats were divided into eight groups, each subjected to varying exposure conditions and OLE treatment. The results indicated that exposure to toluene and noise resulted in significant pulmonary tissue disruption, increased oxidative stress, and elevated levels of pro-inflammatory cytokines. However, OLE administration effectively mitigated these adverse effects, reducing oxidative stress and inflammation and preserving lung histology. This study demonstrates the considerable potential of OLE as a protective agent against environmental pollutants and elucidates its therapeutic potential in preventing pulmonary damage induced by combined exposure to noise and toluene.

## 1. Introduction

In various industrial environments, workers frequently encounter concurrent exposure to multiple stressors. Among these stressors, physicochemical agents such as toluene and noise are known contributors to occupational diseases.

Toluene, an aromatic hydrocarbon solvent extensively used in the industrial and commercial sectors, poses a significant health risk when inhaled. Its metabolic pathways can induce oxidative stress, increasing the production of reactive oxygen species (ROS) [1]. Previous studies have linked toluene exposure to adverse effects on several organs, including the kidneys, liver, and brain [2,3]. Toluene exposure has also been associated with behavioral disorders [4] and adverse cardiac rhythms [5]. Moreover, workers exposed to paint thinners, which often contain elevated levels of toluene, exhibit increased malondialdehyde levels, a byproduct of lipid peroxidation and a marker of oxidative damage [6].

Noise exposure is prevalent across various occupational environments and can lead to sensorineural hearing loss even at levels below legal limits [7,8]. Beyond auditory effects, noise exposure is associated with additional health risks, including an increased likelihood of metabolic disorders, particularly cardiovascular diseases [9,10]. Noise exposure is also linked to sleep disturbances [11], which may compromise cognitive function. Noise exposure elevates ROS levels, including superoxide, hydrogen peroxide, and hydroxyl radicals, contributing to oxidative injury [11].

Concurrent exposure to toluene and noise can significantly exacerbate oxidative stress, which disrupts the balance between ROS production and antioxidant defense. This imbalance frequently increases lipid peroxidation and cellular damage across various tissues, including the heart, brain, and lungs [12,13,14].

The Mediterranean region, known for its diverse plant-based food consumption of mainly fruits, legumes, cereals, and herbs, boasts the olive tree (*Olea europaea* L.), a prominent species in the Oleaceae family. Olive leaves are rich in bioactive polyphenolic substances, including verbascoside, oleuropein, hydroxytyrosol, caffeic acid, and tyrosol [15,16]. Olive leaves are frequently used in human phytotherapy, such as extracts, teas, or powders. They are recognized for their antioxidant, antihypertensive, anti-atherogenic, anti-thrombotic, neurological disorder protection, and cardioprotective activities [17,18]. Polyphenolic compounds found in olive leaves, such as oleuropein, tyrosol, and hydroxytyrosol, have been well-documented for their potent antioxidant capabilities and efficacy in mitigating inflammation [19,20,21]. The antioxidant properties of these compounds are primarily attributed to their structural features, notably the presence and configuration of hydroxyl functional groups in their molecular structure [22]. Furthermore, when these phenolic compounds are combined, they can exhibit synergistic effects, significantly enhancing the antioxidant capacity beyond the cumulative impact of each compound [23].

The primary objective of this study was to comprehensively investigate the protective effects of *Olea europaea* L. leaf extract (OLE) against lung damage induced by exposure to inhaled toluene, noise, or a combination of both. Employing a multidimensional approach, the study included thorough evaluations through histopathological, immunohistochemical (IHC), and biochemical analyses, specifically focusing on cytokine quantification.

## 2. Methods

### 2.1. Preparation of the Olea europaea L. Leaf Extract

The leaves of the ‘Chétoui’ cultivar of *Olea europaea* L. were air-dried before extraction. For the extraction process, a solvent mixture comprising 70% ethanol and 30% water was employed, and the extraction was conducted at 60 °C for 4 h using 5 g of powdered olive leaves [24]. Following extraction, the solution was filtered through a cellulosic filter plate and concentrated under vacuum using a rotary evaporator at 65 °C. The final extract was stored at 4 °C until further use.

### 2.2. HPLC Analysis

HPLC analysis was conducted using a reversed-phase column (Pursuit XRs ULTRA 2.8 (C18, 100 mm × 2 mm) from Agilent Technologies (Stockport, UK). A sample volume of 20 µL was injected, and the column was maintained at 30 °C. The mobile phase consisted of 0.1% formic acid in water (solvent A) and 0.1% in methanol (solvent B). A gradient elution method was implemented, starting with 100% solvent A, gradually shifting to 100% solvent B over 20 min, followed by a 5 min hold at 100% solvent B, and finally returning to 100% solvent A over 25 min. The flow rate was maintained at 1 mL/min throughout the procedure.

### 2.3. Biological Activities of Olea europaea L. Leaf Extract

#### 2.3.1. DPPH Free Radical Scavenging Activity

The DPPH free radical scavenging activity was assessed according to the method described by Ben Abdallah Kolsi [25]. Olive leaf extract concentrations ranging from 5 to 200 µg/mL were prepared precisely. Each concentration was combined with 1 mL of 0.078 mM DPPH, followed by thorough mixing and 30 min incubation without light. Subsequently, the absorbance of the mixture was measured at a 517 nm wavelength. For comparison, ascorbic acid, BHA, and BHT were used as the standard antioxidants. The percentage of DPPH radical inhibition was calculated using the following equation:

Inhibition (%) = [(Abs_control_ − Abs_sample_)]/(Abs_control_)] × 100

where Abs_control_ represents the absorbance of the control solution, and Abs_sample_ denotes the absorbance of the OLE. A linear regression analysis was conducted to determine the IC_50_ value, which indicates the minimum concentration required to neutralize 50% of DPPH radicals. A lower IC_50_ value indicates a higher antioxidant capacity, demonstrating the efficacy of olive leaf extract in scavenging DPPH radicals.

#### 2.3.2. Reducing Power Assay

The reducing potential of olive leaf extract was evaluated using the method described by Ben Abdallah Kolsi [25]. In this procedure, 1 mL of olive leaf extract, prepared at various concentrations (20 to 100 µg/mL), was mixed with 2.5 mL of phosphate buffer (0.2 M, pH 6.6) and 2.5 mL of 1% potassium ferricyanide [K_3_Fe(CN)_6_]. The mixture was then incubated at 50 °C for 20 min. Subsequently, 2.5 mL of 10% trichloroacetic acid was added, and the solution was centrifuged at 3000× *g* for 10 min. From the resulting supernatant, 2.5 mL was combined with 2.5 mL of distilled water and 0.5 mL of freshly prepared 0.1% ferric chloride (FeCl_3_) solution. The optical density of the final mixture was measured at 700 nm, and the reducing power was proportional to the observed absorbance.

#### 2.3.3. Chelating Capacity Assessment of Ferrous Ion

The chelating capacity of ferrous ions in the samples was assessed following the experimental procedure described by Talaz et al. [26]. Briefly, 1.6 mL of the sample at varying concentrations was combined with 1.6 mL of distilled water. To this mixture, 0.4 mL of a FeCl_2_ solution (0.5 mM) was added, and the components were thoroughly homogenized. Subsequently, 0.4 mL of Ferrozine solution (0.5 mM) was introduced, followed by vigorous agitation for 1 min. The solution was allowed to equilibrate at room temperature for 20 min. Absorbance was measured at 562 nm, with blank measurements performed using only the solvent and omitting the sample. The chelation percentage was calculated using the following equation:

%Chelation = [1 − (Abs_562_ sample − Abs_562_ control)] × 100

To determine the IC_50_ value of the sample, a sample curve was constructed based on the absorbance measurements at 562 nm. Ethylene diamine tetra acetic acid (EDTA) was utilized as the standard reference for comparison.

#### 2.3.4. Evaluation of In Vitro Anti-Inflammatory Activity Using the Bovine Serum Albumin (BSA) Denaturation Assay

The approach developed by Williams et al. [27] was employed to investigate the in vitro anti-inflammatory efficacy of OLE. OLE samples with concentrations ranging from 10 to 400 µg/mL were prepared alongside corresponding diclofenac samples. Each sample was combined with 0.05 mL of a 0.5% *w*/*v* bovine serum albumin (BSA) solution and subsequently incubated at 37 °C for 20 min, followed by a 3 min heating period at 57 °C. After cooling to ambient temperature, 2.5 mL of phosphate buffer was added to each solution.

Absorbance measurements at 255 nm were obtained using a spectrophotometer to assess protein denaturation inhibition. A control sample served as a reference, representing 100% protein denaturation. The inhibition of protein denaturation by the OLE was then calculated as a percentage relative to the control, utilizing the following formula:

Inhibition of denaturation (%) = [(Absorbance of control − Absorbance of OLE)/Absorbance of control] × 100

#### 2.3.5. Evaluation of In Vitro Anti-Inflammatory Activity Using the Egg Albumin Denaturation Assay

A 5 mL solution was prepared by mixing 0.2 mL of egg albumin, 2.8 mL of phosphate-buffered saline (PBS, pH 6.4), and 2 mL of olive leaf extract at 10 to 200 µg/mL concentrations. Diclofenac, a standard reference drug, was tested at the same concentrations. For comparison, a control solution containing double the volume of distilled water was used. The mixtures were incubated at 37 °C ± 2 °C for 15 min, followed by heating to 70 °C for 5 min. After cooling, absorbance was measured at 660 nm using the procedure outlined by Chandra et al. [28]. The percentage of inhibition of protein denaturation was calculated using the following formula:

%inhibition = 100 × (Vt/Vc − 1)

In this context, Vt denotes the absorbance of the test sample, while Vc corresponds to the absorbance of the control. The results presented are the mean values derived from three independent replicates.

### 2.4. In Vivo Protocol

**Figure 1:**
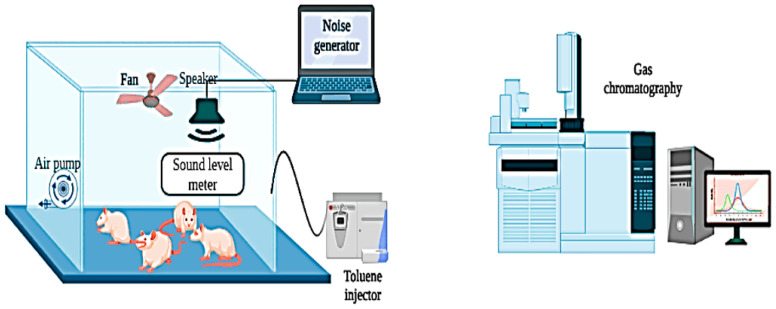
Diagrammatic representation of the experimental configuration for rat exposure to noise and toluene.

This study obtained 48 male *Wistar* rats, aged 4–5 months and weighing 250–300 g, from the Pasteur Institute of Tunis. Rats were randomly assigned to eight groups, with six animals in each group. Before exposure, rats were housed in cages without noise and toluene exposure. Throughout the exposure period, the rats were transferred daily to different rooms for specific exposures: noise exposure (N and N + OLE groups), toluene exposure (T and T + OLE groups), or simultaneous exposure to noise and toluene (NT and NT + OLE groups). The control group (Group C) and the OLE-treated group (Group O) were also transferred to separate chambers located in a noise-free environment with background noise levels consistently maintained below 40 ± 5 dB (A) and devoid of toluene exposure (clean air). This ensured consistent environmental conditions comparable to the other groups. The selection of materials and dimensions for the exposure chamber considered the welfare of the animal subjects and adhered to the specifications of a reverberant chamber. This design guaranteed uniform noise levels within the chamber regardless of distance. Additionally, our recent study employed the comprehensive framework of the general exposure model [12]. Notably, Figure 1, with the author’s permission, provides a visual representation of the exposure chamber, offering insight into the experimental setup and environmental conditions.

The chamber has an internal volume of 0.43 m^3^ and is constructed from plexiglass. Six animals were simultaneously placed in the chamber for a daily exposure period of 6 h, from 8:00 a.m. to 2:00 p.m.

#### 2.4.1. Toluene Exposure Protocol

Two experimental groups, Group T and Group T + OLE, were exposed to toluene (T) for six hours each day, five days a week, over six weeks. The exposure protocol was implemented daily from 8:00 a.m. to 2:00 p.m. in two identical inhalation chambers made of plexiglass. Each chamber was designed to hold six rats simultaneously and was equipped with systems for generation, exposure, and monitoring. The chamber design was adapted from the methodology established by Hinners et al. [29].

To maintain a toluene concentration of 300 ± 10 ppm for six hours in the exposure chamber, liquid toluene (99.5%, Loba Chemie Pvt Ltd., Mumbai, India) was delivered into a mixing box connected to the chamber via an isocratic pump. The chamber featured an inlet fan to promote air circulation and a centrifugal fan at the outlet to regulate adjustable airflow. Toluene levels were continuously monitored and sampled through the circular openings in the chamber. The concentration of toluene was analyzed regularly using gas chromatography. Additionally, the chamber temperature was actively controlled at 23 ± 1 °C, and monitoring was conducted using a digital thermometer to ensure stable experimental conditions [12].

#### 2.4.2. Noise Exposure Protocol

Two experimental groups, Group N and Group N + OLE, were exposed to noise (N) for six weeks. Acoustic stimuli were produced using Audacity 2.3.2, audio editing software. The noise intensity was calibrated to 85 dB (A) within an octave-band noise spectrum ranging from 8 to 16 kHz. A sound speaker was strategically placed to ensure uniform exposure intensity across all the experimental subjects. Continuous monitoring of noise levels during the experiment was conducted using a Class 1 accuracy integrating sound level meter, specifically a Type 2238 Bruel and Kjaer meter (HBK, Nærum, Denmark). Noise exposure sessions were conducted for six hours each day, five days a week, following a predefined schedule from 8:00 a.m. to 2:00 p.m. To maintain a controlled experimental environment, the background noise level in the laboratory was consistently maintained at 40 ± 5 dB (A) [12]. This meticulous control of background noise ensured a standardized experimental setting for investigating the impacts of noise exposure on the experimental groups.

#### 2.4.3. Toluene and Noise Combined Exposure Protocol

To investigate the potential interaction between combined exposure to noise and toluene, two rat cohorts, Group NT and Group NT + OLE, were subjected to concurrent exposure to both toluene and noise over six weeks, with daily exposure lasting six hours. The co-exposure methodology mirrored the procedures employed in previous single-exposure experiments for noise and toluene. However, in this instance, noise and toluene were generated simultaneously, enabling the examination of potential combined effects arising from the simultaneous exposure to these environmental stressors.

#### 2.4.4. Toluene Sampling and Analysis

Preliminary trials were conducted to evaluate toluene concentration in the exposure chamber by introducing a target concentration of 300 ppm. The active sampling method, Method 1501, defined by the U.S. National Institute for Occupational Safety and Health [30], employed a personal sampling air pump (Casella CEL, Apex 182180B-K5, Bedford, UK). Activated charcoal was selected as the adsorbent for vapors from various organic compounds. Following sampling, each segment of the sampler tube was treated with 1 mL of carbon disulfide and agitated for 30 s to ensure complete desorption. Gas chromatography–mass spectrometry was used to analyze the samples (Agilent Technologies 6890N Network GC System, Stockport, UK). This analytical methodology enables precise and reliable measurements of toluene concentrations within the exposure chamber, thus providing critical information for evaluating the experimental conditions and effects of toluene exposure on the study subjects.

### 2.5. Animal Subjects and Experimental Design

Rats were acclimatized in controlled animal housing facilities for one week before the experiments to ensure proper adaptation. The rats were housed in pairs to reduce stress throughout the acclimation and experimental phases. They were given ad libitum access to a commercial pellet diet (Industrial Society of Food, Sfax, Tunisia) and unrestricted drinking water access. The rats were maintained under carefully regulated environmental conditions, including a 12-h light/dark cycle and a room temperature of 23 ± 1 °C.

#### 2.5.1. Ethical Considerations

All experimental protocols were conducted following established guidelines for the care and use of animals. The experiments were approved by the Ethics Committee of the National School of Veterinary Medicine of Sidi Thabet (reference: 162020/FMT). This approval was consistent with the framework established in a recent study by Ben Attia et al. [12] and complied with the International Council for Laboratory Animal Science (ICLAS) recommendations.

#### 2.5.2. Group Allocation and Experimental Design

A total of 48 male *Wistar* rats were allocated into 8 groups comprising 6 animals each as follows:

C Group: Control rats housed under standard conditions.

O Group: Rats exclusively administered OLE at 40 mg/kg/day, daily at 7:00 a.m., for 6 weeks.

Group N: Rats subjected solely to noise exposure.

N + OLE Group: Rats exposed to noise and administered OLE at 40 mg/kg/day, daily at 7:00 a.m., for 6 weeks.

T Group: Rats subjected solely to toluene exposure.

T + OLE Group: Rats exposed to toluene and administered OLE at 40 mg/kg/day, daily at 7:00 a.m., for 6 weeks.

NT Group: Rats co-exposed to both noise and toluene.

NT + OLE Group: Rats simultaneously exposed to both toluene and noise and administered OLE at 40 mg/kg/day, daily at 7:00 a.m., for 6 weeks.

#### 2.5.3. Justification for Selecting Noise Intensity, Toluene Concentration, and *O. europaea* L. Leaf Extract Dosage

In a recent epidemiological investigation by Ben Attia et al. [31], the combined effects of noise and solvent exposure within a specialized manufacturing facility for composite products in the sports sector were explored. The findings indicated that toluene was the primary solvent used, consistently maintaining a concentration of 300 ppm, whereas an ambient sound intensity of 85 dB (A) was observed at various workstations. This research significantly enhances our understanding of the health risks of simultaneous exposure to multiple hazardous substances in occupational settings, impacting occupational health and safety regulations.

A pivotal study conducted by Bahri et al. [32] at the Experimental Medicine Unit of the Faculty of Medicine in Tunis determined the dosage of *O. europaea* L. leaf extract. This study assessed three different doses of the extract (10, 20, and 40 mg/kg/day) and determined that the highest dose of 40 mg/kg/day significantly improved the management of pulmonary fibrosis. Identification of this optimal dosage is crucial for guiding subsequent research.

Furthermore, the 40 mg/kg dose evaluation encompassed a comprehensive examination of its cardioprotective properties in *Wistar* rats exposed to noise, toluene, or a combination of both. As detailed in a recent study by Ben Attia et al. [12], the results underscored the extract’s potential cardioprotective effects, mitigating the adverse impacts associated with isolated or simultaneous exposure to toluene and noise.

### 2.6. Sample Collection

Following the completion of the exposure period, the rats were anesthetized with ketamine hydrochloride (50 mg/kg) and euthanized via decapitation. Lung tissues were promptly excised and washed with 0.1 M phosphate-buffered saline (pH 6.8). The tissues were divided into two segments for histopathological and immunohistochemical examinations and analysis of oxidative stress markers.

### 2.7. Macroscopic Observation

For the macroscopic observation of rat lungs, euthanasia was performed following approved methods. The chest cavity was then opened, and the lungs were carefully extracted and positioned on a sterile dissection tray. Utilizing a stereomicroscope, observations were conducted to identify any macroscopic changes, including alterations in color, size, and shape. Additionally, the lungs were thoroughly examined for signs of lesions, congestion, or inflammation. All observed anomalies were meticulously documented. Notably, the macroscopic observations were carried out by two trained researchers blinded to the treatment groups, mitigating potential biases in the assessment process.

### 2.8. Oxidative Stress Markers Determination

The lung tissue was homogenized using the T 25 digital Ultra-Turrax^®^ apparatus (IKA-Werke GmbH & Co. KG, Staufen, Germany) in ice-cold PBS buffer (pH = 7.2). After homogenization, the mixture was centrifuged for 20 min at 10,000 rpm at 4 °C. The supernatant obtained post-centrifugation was then divided into aliquots and stored at −80 °C for subsequent use.

#### 2.8.1. Lipid Peroxidation Determination

Lipid peroxidation levels were evaluated according to the protocol established by Buege and Aust [33]. The samples were treated with a butylated hydroxytoluene/trichloroacetic acid (BHT–TCA) solution at varying concentrations ranging from 1 to 20% (*v*/*v*) and then centrifuged at 850× *g* for 10 min. The resulting supernatant was combined with a tertiary butyl alcohol (TBA)–Tris solution (120 mM–26 mM, *v*/*v*) under acidic conditions (0.6 M HCl). The mixture was heated at 100 °C for 15 min and cooled to room temperature. Absorbance was measured at 532 nm using a UV-spectrophotometer (Labomed, UV-2650, Inc., Los Angeles, CA, USA), and the results were quantified as nmol of MDA/mg of protein.

#### 2.8.2. Assessment of Catalase Activity

Catalase (CAT) activity was evaluated according to the methodology described by Aebi et al. [34]. This assay is predicated on the enzyme’s ability to catalyze the decomposition of hydrogen peroxide (H_2_O_2_), which results in a measurable decline in the absorbance of the reaction mixture at 240 nm. Catalase activity was expressed in μmoles/min/mg of protein.

#### 2.8.3. Assessment of Superoxide Dismutase (SOD) Activity

Superoxide dismutase (SOD) activity was measured using the methodology established by Beyer and Fridovich [35]. This assay used epinephrine in 50 mM carbonate buffer (pH 10.2) in conjunction with bovine catalase (0.4 U/mL). The underlying principle of this method is predicated on the inhibition of the auto-oxidation of epinephrine to adrenochrome by SOD. Absorbance was measured at 480 nm, and SOD activity was quantified and expressed in UI/min/mg of protein.

#### 2.8.4. Total Protein Level Determination

The protein concentration in lung samples was quantified using the Bradford assay [36], employing bovine serum albumin (BSA) as a standard reference. Absorbance measurements were taken at 595 nm, and the total protein content was calculated and expressed in milligrams per milliliter.

### 2.9. Histological Study

To prepare the lung tissues for histological examination, the tissue underwent perfusion and immersion in a fixative solution (10% neutral-buffered formalin) for 48 h. After fixation, the lungs were dehydrated using ethanol in a graded series, followed by embedding in paraffin. Sections of 4–5 μm thickness were then obtained using a microtome (Leica Biosystems RM2245, Leica Biosystems, Milton Keynes, UK). These sections were stained with hematoxylin and eosin (H&E) for subsequent microscopic analysis.

### 2.10. Immunohistochemistry Study

Lung tissues were initially fixed in 10% neutral-buffered formalin, embedded in paraffin, and sectioned at a thickness of 5 μm. The paraffin-embedded sections underwent dewaxing through two sequential immersions in toluene for 5 min each and rehydrated using graded ethanol concentrations (100%, 90%, and 70%) for two cycles of 3 min. The sections were then immersed in distilled water for 5 min, treated with a citric buffer solution (0.01 M, pH 6) under microwave irradiation at 300 W for 20 min, cooled at room temperature, and rinsed three times with Tris-buffered saline (TBS, 50 mM, pH 7.6) for 5 min each. To detect the S100A9 antibody, the Novolink Polymer Detection System (Leica Biosystems, Newcastle upon Tyne, UK) was applied following the manufacturer’s guidelines. The sections were incubated with 3,3′-diaminobenzidine (DAB) solution for 30 s, followed by a rapid rinse in TBST solution, immersion in hematoxylin, water rinsing, and eosin staining. Dehydration was achieved using two 5-min baths each in 95% and 100% ethanol, followed by three consecutive toluene baths for 5 min each. Negative controls were incorporated by substituting the primary antibody with nonimmune immunoglobulins. The prepared sections were analyzed using a DM 1000 Leica photomicroscope (Leica Microsystems, Milton Keynes, UK), and macrophage cell counts were conducted in 100 fields per group. The results were reported as the number of labeled cells per mm^2^.

### 2.11. Measurement of Pro-Inflammatory Parameters in the Serum

Serum concentrations of interleukin-6 (IL-6), tumor necrosis factor-alpha (TNF-α), and interleukin-1β (IL-1β) were quantified using an enzyme-linked immunosorbent assay (ELISA). Specific rat ELISA kits were employed, including Rat SimpleStep ELISA kits for IL-6 (ab234570, Abcam, Cambridge, UK), IL-1β (ab100767, Abcam, Cambridge, UK), and TNF-α (ab46070, Abcam, Cambridge, UK). All assays were conducted using the manufacturer’s protocols to ensure precise and reliable quantification of these cytokines in serum samples.

### 2.12. Statistical Analyses

The results are presented as mean values with corresponding standard deviations (mean ± SD). Statistical analyses were conducted using the Biostat software 5.3.0 for Windows. Group differences (n = 6) were evaluated using the Kruskal–Wallis and Mann–Whitney U tests for post-hoc multiple comparisons. A *p*-value less than 0.05 (*p* < 0.05) was deemed statistically significant.

## 3. Results

### 3.1. Profiling and Identification of Bioactive Molecules in O. europaea Leaf Extract

**Figure 2:**
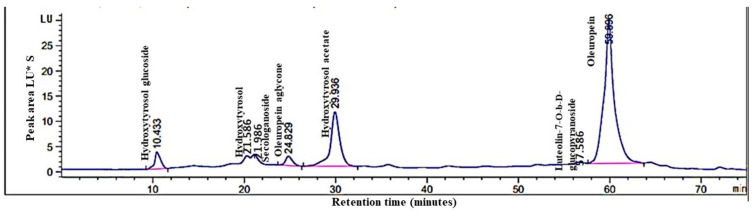
High-performance liquid chromatography (HPLC) analysis of phenolic compounds in *O. europaea* L. leaf extract: chromatographic profile.

Phenolic compounds in olive leaf extract analysis using high-performance liquid chromatography (HPLC) (Figure 2) successfully identified seven distinct compounds: hydroxytyrosol glucoside (retention time: 10.433 min), hydroxytyrosol (21.586 min), secologanoside (21.986 min), oleuropein aglycone (24.829 min), hydroxytyrosol acetate (29.936 min), luteolin-*O*-β-d-glucopyranoside (57.586 min), and oleuropein (59.896 min).

### 3.2. Extract In Vitro Antioxidant Efficacy of O. europaea Leaf Extract

The antioxidant activity of *O. europaea* leaf extract (OLE) was assessed through various established tests, as summarized in Table 1. Initially, the DPPH radical scavenging assay was used to gauge the antioxidant potential of the extract. The IC_50_ value, representing the median inhibitory concentration, served as a quantitative measure, with a lower value indicative of higher antioxidant efficacy. Our study revealed a notable IC_50_ value of 13.5 µg/mL for OLE, suggesting significant antioxidant activity. In comparison, the IC_50_ values of the reference compounds BHT, BHA, and ascorbic acid were 8, 4.32, and 2.5 µg/mL, respectively.

Subsequently, the reducing power of OLE was examined by assessing its ability to convert potassium ferricyanide (Fe(CN)_6_)^3−^ to potassium ferrocyanide (Fe(CN)_6_)^4−^. The results exhibited a remarkable reducing power of approximately 73.4 ± 4.08 µg/mL for OLE, underscoring its robust antioxidant properties.

Lastly, the chelating activity test was conducted to further validate OLE’s antioxidant potential. The results demonstrated a chelating activity of approximately 25.53 ± 2.58 µg/mL, providing additional evidence of its antioxidant efficacy.

### 3.3. In Vitro Efficacy of Olea europaea Leaf Extract for Anti-Inflammatory Activity

The anti-inflammatory activity of *Olea europaea* leaf extract (OLE) was systematically investigated using two in vitro methods: the BSA denaturation assay and the egg albumin denaturation method.

The inhibitory effects of OLE and diclofenac, a standard reference drug, were evaluated in the BSA denaturation assay. At a concentration of 400 µg/mL, OLE demonstrated a remarkable maximum inhibition and denaturation effect of 98.32 ± 0.41%, surpassing the notable 97.98 ± 0.01% inhibition achieved by diclofenac at the same concentration (Table 2). These results indicate that OLE possesses potent anti-inflammatory properties, exhibiting superior efficacy compared to diclofenac in the BSA denaturation assay.

Subsequently, the anti-inflammatory potential of OLE was assessed using the egg albumin denaturation method. Testing OLE at a 200 µg/mL concentration exhibited an impressive 99.61 ± 0.67% inhibition of egg albumin denaturation, highlighting its anti-inflammatory solid activity (Table 3). Although diclofenac displayed a slightly higher inhibition of 99.73 ± 0.30% at the same concentration, the difference was marginal, indicating comparable anti-inflammatory efficacy between OLE and diclofenac in this assay.

### 3.4. Olea europaea Leaf Extract Preserves Antioxidant Activity and Mitigates Lipid Peroxidation in Lung Tissues Due to Noise and Toluene Exposure

**Figure 3:**
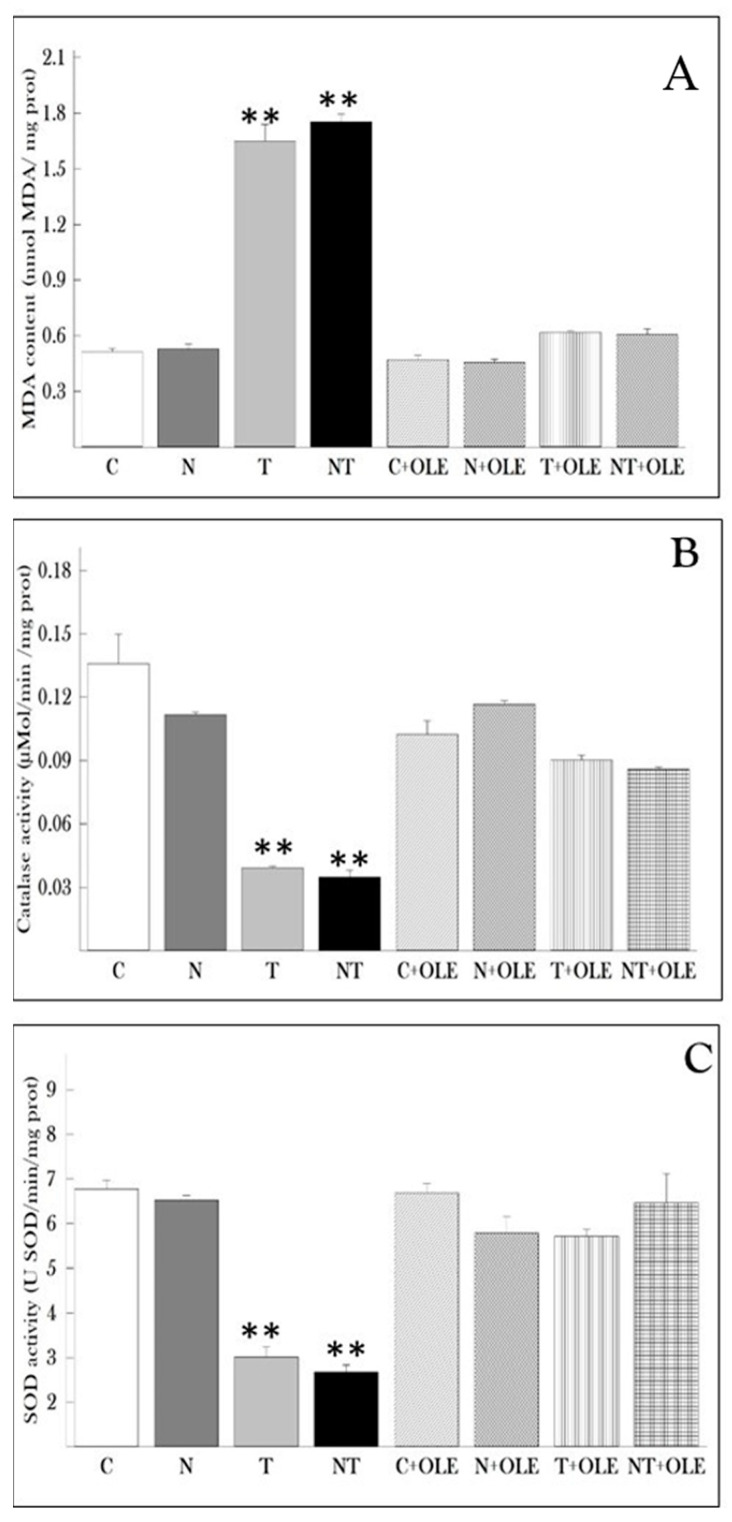
Protective effect of *Olea europaea* leaf extract administration on lung oxidative stress parameters in rats. (**A**) Malondialdehyde (MDA) levels, (**B**) catalase (CAT) activity, and (**C**) superoxide dismutase (SOD) activity. Data are presented as the mean ± SD for six animals per group. Statistical analysis was performed using the Mann–Whitney U test, with ** *p* ≤ 0.01 considered statistically significant compared to the control group.

Figure 3 depicts the results of the MDA assay, along with SOD and CAT activities in lung tissues. The MDA assay aimed to gauge lipid peroxidation levels in the lungs of rats exposed to different conditions (Figure 3A). The results unveiled a substantial increase in MDA levels in the lung tissues of rats exposed to toluene (T) and co-exposed to noise and toluene (NT) compared to the control group (1.64 ± 0.258 vs. 0.513 ± 0.055 nmol/mg protein and 1.752 ± 0.138 vs. 0.513 ± 0.055 nmol/mg protein, *p* ≤ 0.01, respectively). Notably, rats treated with *Olea europaea* L. leaf extract exhibited no significant difference in MDA levels compared to the control group.

Moreover, catalase activity was evaluated in lung tissues (Figure 3B). The results demonstrated that treatment with *Olea europaea* L. leaf extract maintained stable levels of catalase activity in the lung tissues of rats. Conversely, exposure to toluene (T) and co-exposure to noise and toluene (NT) significantly reduced catalase activity compared to the control group (0.039 ± 0.003 vs. 0.135 ± 0.04 and 0.034 ± 0.009 vs. 0.135 ± 0.04, *p* < 0.01, respectively).

Furthermore, the study revealed a significant decrease in the activity of superoxide dismutase (SOD) in rats exposed to toluene alone or in combination with noise compared to the control group (*p* ≤ 0.01). Specifically, rats co-exposed to noise and toluene exhibited a significant decrease in SOD activity (2.677 ± 0.489 U SOD/min/mg of protein) compared to rats exposed to toluene alone (3.012 ± 0.663 U SOD/min/mg of protein) and control rats (6.77 ± 0.627 U SOD/min/mg of protein). Interestingly, exposure to noise alone did not impact SOD activity in lung tissue. However, treatment with OLE significantly restored SOD activity compared to the control group (Figure 3C).

### 3.5. Olea europaea Leaf Extract Preserves Lung Tissue Against Damage from Toluene and Noise Exposure

**Figure 4:**
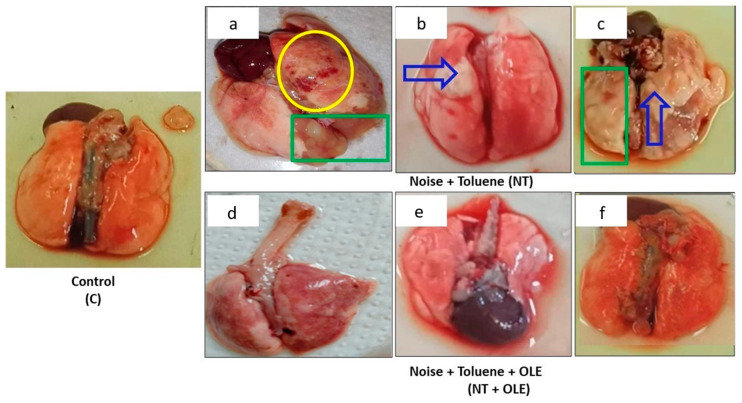
Macroscopic finding in the lung labeled (C) indicates the lung from the control group, (**a**–**c**) shows the lung from the group of rats exposed to noise and toluene, and (**d**–**f**) identifies the lung from the group of rats exposed to noise and toluene receiving OLE. The yellow circle indicates localized foci of hemorrhage, the green rectangle indicates areas of pulmonary edema, and the solid blue arrow indicates the presence of a pulmonary abscess.

Macroscopic examination revealed localized foci of hemorrhage, lung edema, and pulmonary abscess in both the toluene-exposed (T) and the toluene and noise co-exposed (NT) groups, compared to the control group (C) (Figure 4a–c). Notably, the OLE treatment normalized lung appearance, resembling that of the control group (C) (Figure 4d–f).

**Figure 5:**
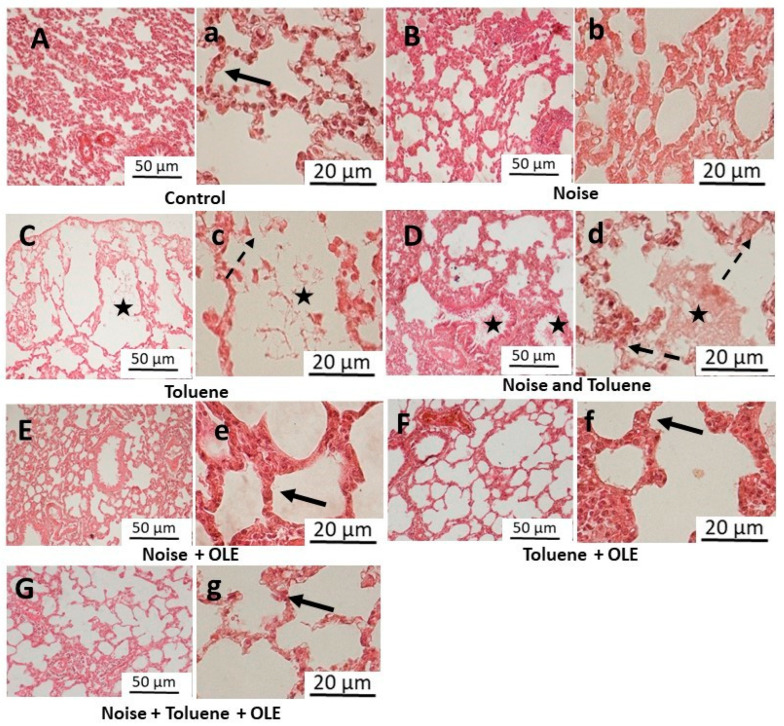
Representative light microscopic images of lung tissue sections (H&E stain) following exposure to noise and/or inhalation of toluene and the protective effect of *Olea europaea* L. leaf extract. (**A**–**G**) (×10 objective), the scale bar indicates 50 μm, and (**a**–**g**) (×40 objective), the scale bar indicates 20 μm. The solid black arrow indicates intact alveolar epithelium, whereas the dashed black arrow indicates the regions exhibiting damage and fragmentation of the alveolar epithelium. The asterisk symbol denotes the presence of parenchymal debris within the interalveolar and intrabronchial spaces.

Histopathological evaluation of lung tissues (Figure 5) in the control group (C) showed normal lung tissue with uniform alveoli and intact alveolar epithelium. Noise exposure (N) did not cause significant changes in lung histology, maintaining an intact appearance and preserved interalveolar septa similar to the control group (C). In contrast, rats exposed to toluene (T) and those co-exposed to toluene and noise (NT) exhibited parenchymal debris, pulmonary edema, and alveolar septa fragmentation, along with the obstruction of numerous alveoli.

OLE treatment improved lung histology, restoring normal observations in all groups. The significant reduction in edema and preserved alveolar septa in OLE-treated groups resembled the control group (C), suggesting a protective effect on lung tissue and mitigating the adverse effects of toluene exposure.

### 3.6. Protective Effects of Olea europaea Leaf Extract on Macrophage Response in Rat Lung Tissues Exposed to Toluene and Noise

**Figure 6:**
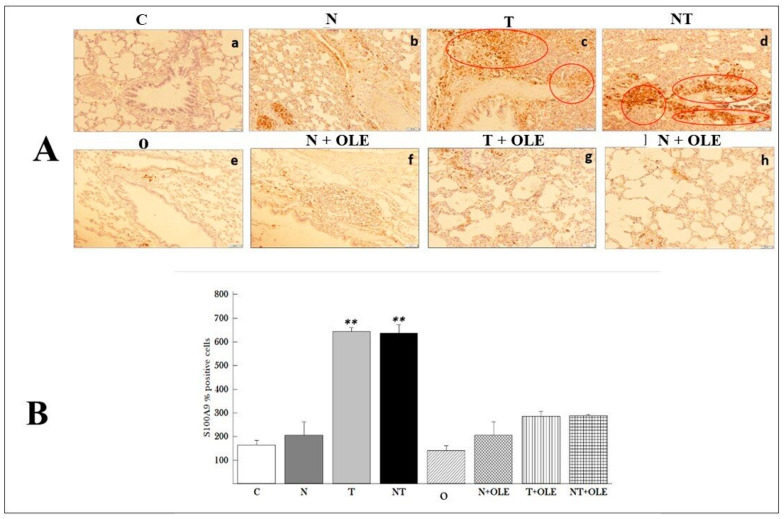
Immunohistochemical staining of the S100A9 macrophage marker in pulmonary tissues and quantitative analysis. (**A**) Representative immunohistochemistry of S100A9 for detecting macrophage level in rat lungs revealed brown areas in pulmonary parenchyma (red circle); (**a**–**h**) (×10 objective), the scale bar indicates 50 μm. (**B**) Number of macrophages counted in 100 fields per group and expressed per mm^2^. Results are expressed as means ± SD of six animals. Different pairs of groups were compared using the Mann–Whitney U test. ** *p* ≤ 0.01 vs. control group. (C, n = 6) Control group; (N, n = 6) Noise exposure group; (T, n = 6) Toluene exposure group; (NT, n = 6) Combined noise and toluene exposure group; (O, n = 6) OLE-treated group; (N + OLE, n = 6) Noise exposure with OLE treatment group; (T + OLE, n = 6) Toluene exposure with OLE treatment group; (NT + OLE, n = 6) Combined noise and toluene exposure with OLE treatment group.

Figure 6 depicts the results of the immunohistochemical analysis of the S100A9 marker, which is used to identify macrophages in rat lung tissues. Immunohistochemical staining revealed brown areas in the lung parenchyma (red circle), indicating the presence of macrophages in the samples under examination.

The immunohistochemical analysis results indicated that noise exposure did not impact the number of labeled cells (Figure 6A(b)). In contrast, exposure to toluene (Figure 6A(c)) or co-exposure to noise and toluene (Figure 6A(d)) led to a significant increase in labeled cells. In contrast, the number of labeled cells in the groups of rats receiving *Olea europaea* L. leaf extract (T + OLE and NT + OLE) showed enhancement (Figure 6A(g,f)).

Quantitatively, the study revealed a significant rise in the number of macrophages counted in 100 fields per group in rats exposed to toluene and co-exposed to toluene and noise compared to the control group (643.66 ± 49.5 vs. 190.67 ± 7.25 and 652.86 ± 49.5 vs. 190.67 ± 27.25, *p* < 0.01, respectively).

### 3.7. Anti-Inflammatory Potential of Olea europaea Leaf Extract: Reduction in Serum TNF-α, IL-1β, and IL-6 Levels Following Noise and Toluene Exposure

**Figure 7:**
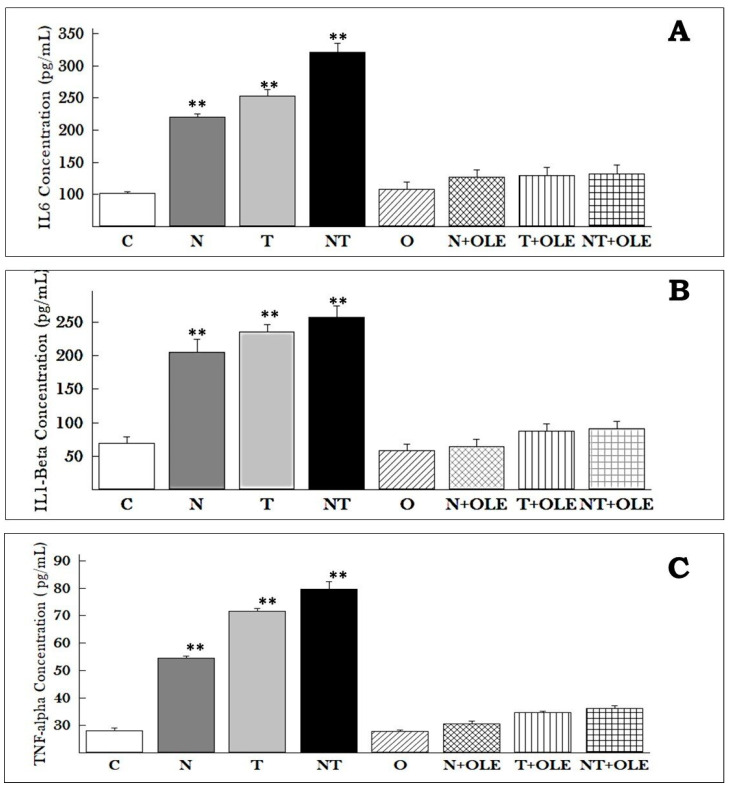
IL-6, IL-1β, and TNF-α concentrations in the serum of *Wistar* rats. The results, presented as means ± SD of six animals, underwent statistical analysis using the Mann–Whitney U test. ** *p* ≤ 0.01 vs. the control group. Cytokine concentrations in pg/mL for IL-6 (**A**), IL-1β (**B**), and TNF-α (**C**) are illustrated. OLE supplementation effectively maintains IL-6, IL-1β, and TNF-α levels compared to the control group, even amidst noise and/or toluene exposure.

The pro-inflammatory cytokines TNF-α, IL-1β, and IL-6 in the serum (pg/mL) were quantified, as illustrated in Figure 7. The investigation revealed a pronounced increase in pro-inflammatory cytokine concentrations in the serum due to noise exposure (N), toluene (T), and combined exposure to noise and toluene (NT) compared to the control group (C). Specifically, the levels of TNF-α increased significantly in all three exposure groups as compared to the control group: 54.5 ± 1.81 pg/mL versus 28.16 ± 2.04 pg/mL (*p* ≤ 0.05) for the N group, 71.5 ± 2.81 pg/mL versus 28.16 ± 2.04 pg/mL (*p* ≤ 0.01) for the T group, and 79.66 ± 6.80 pg/mL versus 28.16 ± 2.04 pg/mL (*p* ≤ 0.01) for the NT group. Similarly, the levels of IL-6 showed a substantial increase in the N, T, and NT groups compared to the control group: 219.62 ± 10.66 pg/mL, 252.52 ± 92.94 pg/mL, and 341 ± 14.01 pg/mL versus 101.16 ± 8.17 pg/mL, respectively (*p* ≤ 0.01). Additionally, the levels of IL-1β were significantly higher in the N, T, and NT groups compared to the control group: 205.5 ± 19.36 pg/mL, 235.5 ± 11.16 pg/mL, and 257.83 ± 16.82 pg/mL versus 68.83 ± 9.49 pg/mL, respectively (*p* ≤ 0.01).

Notably, supplementation with OLE significantly preserved the levels of pro-inflammatory mediators in the exposure groups treated with OLE—noise (N + OLE), toluene (T + OLE), and combined noise and toluene (NT + OLE)—compared to rats co-exposed to noise and toluene (NT).

TNF-α levels were found to be 30.53 ± 2.43 pg/mL, 34.52 ± 1.22 pg/mL, and 36.23 ± 2.09 pg/mL in the N + OLE, T + OLE, and NT + OLE groups, respectively, compared to 79.66 ± 6.80 pg/mL in the NT group (*p* ≤ 0.01). IL-1β levels were 64.58 ± 10.77 pg/mL, 87.16 ± 11.03 pg/mL, and 90.63 ± 11.01 pg/mL in the N + OLE, T + OLE, and NT + OLE groups, respectively, compared to 257.83 ± 16.82 pg/mL in the NT group (*p* ≤ 0.01). IL-6 levels were found to be 127.23 ± 10.96 pg/mL, 129.66 ± 12.20 pg/mL, and 132.15 ± 13.08 pg/mL in the N + OLE, T + OLE, and NT + OLE groups, respectively, compared to 321 ± 14.01 pg/mL in the NT group (*p* ≤ 0.01).

These findings suggest that OLE has a protective effect against the inflammatory response induced by environmental stressors such as noise and toluene. The significant decrease in cytokine concentrations indicated the potential of OLE as a potent anti-inflammatory agent.

## 4. Discussion

Exposure to physical stressors, including noise and industrial chemicals such as toluene, is well recognized to have detrimental health effects [37]. Toluene, when inhaled, predominantly permeates the lungs, rendering them particularly vulnerable to toluene-induced damage [38]. This study aimed to evaluate the deleterious effects of exposure to noise, toluene, or a combination thereof on pulmonary tissue, with particular emphasis on examining the potential protective properties of *O. europaea* leaf extract (OLE). Employing a multifaceted approach, including histopathological, immunohistochemical (IHC), and biochemical analyses, we intend to gauge the extent of damage inflicted by these environmental stressors on lung tissue. The hypothesis is that with its potent antioxidant and anti-inflammatory properties, OLE could provide significant protection against the harmful effects of toluene and noise exposure on the lungs.

*Olea europaea* leaves have a longstanding history of medicinal use, attributed to their remarkable antioxidant and anti-inflammatory properties. Abundant in polyphenols, including oleuropein, hydroxytyrosol, and tyrosol, these leaves offer myriad health benefits. Additionally, the presence of flavonoids, specifically rutin and quercetin, further contributes to the health-promoting attributes of olive leaves, with associations with anti-cancer properties and neuroprotective effects against degenerative diseases [39,40,41]. The rich polyphenolic and flavonoid content of *Olea europaea* leaves positions them as a valuable natural resource, showcasing their potential in promoting human health and as a preventive measure against chronic diseases.

The process of protein denaturation emerges as a pivotal initiator of inflammatory responses [42]. Interestingly, research has revealed that non-steroidal anti-inflammatory drugs not only serve to inhibit pro-inflammatory prostaglandins but also impede the process of protein denaturation [43]. In the scope of our investigation, we conducted a thorough examination to elucidate the impact of *Olea europaea* leaf extract on the dynamics of protein denaturation. This exploration is a critical component of our efforts to understand the potential anti-inflammatory mechanisms of *Olea europaea* leaf extract, shedding light on its multifaceted influence on cellular processes.

At a 400 µg/mL concentration, OLE showcased an impressive denaturation inhibition of 98.31%, resulting in substantial significance. Concurrently, diclofenac exhibited a slightly lower but still noteworthy 97.98% inhibition against bovine serum albumin (BSA) denaturation under identical conditions. In our pursuit of understanding the anti-inflammatory capabilities of OLE, we employed the egg albumin denaturation technique, setting diclofenac as a benchmark and probing OLE at a concentration of 200 µg/mL. Remarkably, OLE demonstrated a remarkable 99.61% inhibition of egg albumin denaturation, highlighting its potent anti-inflammatory activity. Notably, while diclofenac exhibited a slightly higher inhibition of 99.73% at the equivalent concentration, the difference proved marginal, indicating a comparable anti-inflammatory efficacy between OLE and diclofenac in this specific assay. These findings illuminate the anti-inflammatory potential of *Olea europaea* leaf extract, positioning its effectiveness on par with the well-established anti-inflammatory agent diclofenac. Furthermore, our observations align with the prior literature [42,43], reinforcing the extract’s potential as a promising avenue for inflammation interventions.

Numerous studies have consistently underscored the antioxidant potential of olive leaves, underscoring the potency of key polyphenolic compounds, notably oleuropein, hydroxytyrosol, and tyrosol [24,44]. The substantial concentration of polyphenols and flavonoids within olive leaves contributes significantly to their robust antioxidant properties. This attribute positions them as a valuable natural reservoir, well-suited for promoting human health and serving as a preventive measure against chronic diseases [45]. The cumulative evidence from these studies affirms olive leaves’ significant antioxidant capacity, emphasizing their potential to contribute to overall well-being and mitigate the risk of various long-term health conditions.

In our investigation, we meticulously evaluated the antioxidant potential of OLE through the DPPH free radical scavenging assay. The results unveiled OLE’s notable antioxidant efficacy, as evidenced by an IC_50_ value of 13.5 µg/mL. This aligns seamlessly with previous research affirming the robust antioxidant properties of olive leaf extracts [45,46]. Furthermore, the ferric reducing antioxidant power (FRAP) assay substantiated the significant reducing power of OLE, measured at approximately 73.4 ± 4.08 µg/mg. This heightened reducing power is attributed to the electron-donating capacity of the polyphenol content, as documented by Guimaraes et al. [47]. These findings collectively emphasize the remarkable antioxidant prowess of OLE, reinforcing its potential as a valuable natural resource with significant implications for oxidative stress mitigation and overall health promotion.

Our investigation revealed a clear and positive correlation between the polyphenolic content of *Olea europaea* leaf extract and its antioxidant properties. The reductive agents employed facilitated the conversion of ferricyanide-Fe^3+^ complexes into the ferrous Fe^2+^ form, contributing significantly to the observed antioxidant activity [48]. Recognizing antioxidants as both reducers and oxidant inactivators, our findings underscore the pivotal role of reducing capacity in determining the potential antioxidant efficacy of the extract, which is in line with the insights provided by Bourgou et al. [49]. Additionally, our assessment included examining the extract’s chelation ability, which revealed a notable capacity (approximately 25.53 ± 2.58 µg/mL) to neutralize reactive metal ions and thereby mitigate oxidative stress. 

Our findings highlight the significant antioxidant potential inherent in olive leaf extract. Diverse phenolic compounds in OLE, including oleuropein, tyrosol, and hydroxytyrosol, contribute to its antioxidant activity through their unique structural attributes [22]. It is important to note that considerations regarding sample sources, extraction methods, and analytical protocols play a pivotal role in ensuring the accurate and precise evaluation of antioxidant properties [43,50]. This acknowledgment underscores the importance of standardized procedures in OLE research to yield reliable and comparable results, thus enhancing the credibility of studies investigating its antioxidant capabilities.

Oxidative stress is critical in the pathogenesis of various respiratory diseases and can exacerbate the inflammatory response. Investigating oxidative stress biomarkers becomes essential, especially in toluene and noise exposure, explicitly focusing on lung tissue. Oxidative stress has been identified as a significant contributor to pulmonary tissue damage in various diseases [51]. Therefore, exploring the potential protective effects of olive leaf extract on lung tissue is imperative, specifically in mitigating the harmful impacts induced by toluene and noise exposure. This exploration, conducted through the assessment of oxidative stress biomarkers, holds substantial importance in understanding the underlying mechanisms and potential interventions for safeguarding respiratory health in occupational settings.

In this context, toluene has been documented to induce the production of reactive oxygen species (ROS) production during its metabolic processing, adversely affecting multiple organs, including the liver, lungs, kidneys, and various brain regions [52,53]. The present study indicated a significant increase in lipid peroxidation within the lung tissue of rats exposed to toluene and in those co-exposed to noise and toluene. Elevated lipid peroxidation is a prevalent consequence of human oxidative stress after exposure to toxic agents. Notably, inhalation of toluene has been associated with substantial increases in blood and tissue concentrations of malondialdehyde (MDA), along with a concurrent decline in antioxidant enzyme activity [53].

Moreover, simultaneous exposure to noise and toluene appears to adversely influence the antioxidant status in rat lung tissues, potentially resulting in reduced catalase (CAT) and superoxide dismutase (SOD) activities. Abouee-Mehrizi et al. [54] highlighted significant alterations in the oxidant–antioxidant balance in the kidneys and liver attributable to exposure to noise and toluene, which caused disturbances in the activity of glutathione peroxidase (GPx) and SOD, as well as fluctuations in MDA levels.

It is widely acknowledged that oxidative stress is a fundamental mechanism underlying the toxicity associated with volatile organic compounds. This toxicity is characterized by an imbalance between ROS generation and antioxidant defense mechanisms. This understanding further emphasizes the complex interplay between concurrent exposure to noise and toluene and the resultant oxidative stress, particularly regarding potential respiratory health consequences.

The current study demonstrates that treatment with OLE effectively mitigated the disruptions in oxidative stress markers caused by exposure to toluene and noise in rat lung tissue. OLE, derived from the leaves of *Olea europea* L., is known to contain a wide variety of bioactive compounds, including phenolic acids, flavonoids, secoiridoids, and triterpenes, all of which possess potent antioxidant properties [15,16]. Among the key constituents of OLE are oleuropein and hydroxytyrosol, recognized for their ability to neutralize free radicals, reduce lipid peroxidation, and enhance the activity of antioxidant enzymes like catalase and SOD [55,56,57]. Furthermore, OLE contains other phenolic compounds such as verbascoside, luteolin-7-*O*-glucoside, and apigenin-7-*O*-glucoside, each contributing to robust antioxidant activities [58].

The high content of bioactive compounds in OLE plays a crucial role in alleviating oxidative stress, providing a plausible explanation for its therapeutic efficacy in our study. These results imply that OLE holds promise as a therapeutic intervention for ameliorating oxidative stress induced by exposure to toluene and noise in industrial settings. Overall, the rich composition of OLE, particularly its abundance of bioactive compounds with potent antioxidant properties, makes it a promising therapeutic option for reducing oxidative stress and associated health complications.

It is noteworthy that the (N) group did not exhibit any significant histological alterations, unlike the (T) or (NT) rats, which showed considerable damage to lung architecture. Exposure to volatile organic compounds, such as toluene, can initiate respiratory tract irritation and inflammation, potentially leading to severe pulmonary damage and even cancer [52]. Remarkably, treatment with OLE effectively improved lung histology in the rats that received it. Our findings showed a substantial reduction in edema and parenchymal debris, while alveolar septa were preserved similarly to the control group. This suggests the potential therapeutic benefits of OLE against lung damage induced by exposure to toluene and noise.

To comprehensively assess the inflammatory response in lung tissue following exposure to toluene and noise, an immunohistochemistry (IHC) assay was conducted to examine the abundance of pulmonary macrophages. S100A9, a calcium-binding protein belonging to the S100 family, exhibits high expression in immune cells such as monocytes and macrophages and plays a pivotal role in acute and chronic inflammation. Its involvement has been documented in various inflammatory diseases, including rheumatoid arthritis, inflammatory bowel disease, and several types of cancer [59].

In the context of lung inflammation induced by toluene and noise exposure, S100A9 emerges as a valuable marker for detecting pulmonary macrophages, pivotal contributors to the immune response in the lungs. Immunohistochemistry targeting S100A9 expression in lung tissue offers valuable insights into the extent of macrophage infiltration and the severity of lung inflammation. Gebhardt et al. [60] demonstrated that S100A9 is a crucial pro-inflammatory mediator in acute and chronic inflammation, which plays a central role in macrophage recruitment and activation. Furthermore, S100A9 is implicated in the regulation of cytokine production and the activation of key signaling pathways, including NF-κB and MAPK, known to play roles in the pathogenesis of inflammation [61].

In this study, we investigated the impact of toluene exposure and the combined exposure to toluene and noise on pulmonary macrophages, crucial components in the innate defense of the respiratory tract. Our results showed that both toluene exposure alone and concurrent exposure to noise and toluene increased pulmonary macrophage levels. This dysregulation has the potential to contribute to pulmonary emphysema, causing airway obstruction and damage to the alveolar wall. We propose that inhaling toluene, particularly with exposure to a physical agent like noise, might induce the recruitment of various inflammatory cells in the respiratory tract, promoting innate and adaptive immune responses.

In light of recent studies highlighting the potential of *Olea europaea* L. leaf extract (OLE) as a natural anti-inflammatory agent [62,63,64], we sought to explore the impact of OLE administration on pulmonary macrophages in rats exposed to toluene and noise. Our findings revealed a significant reduction in the number of pulmonary macrophages following OLE treatment. This suggests a potential role for OLE in alleviating inflammation induced by noise and toluene exposure in the lungs.

Our findings are consistent with previous studies, such as those of Kim et al. [65], which demonstrated the inhibitory impact of oleuropein, a prominent bioactive compound in OLE, on macrophage infiltration in mice. This anti-inflammatory effect of OLE could be attributed to its capacity to modulate signaling pathways integral to the immune response, as proposed by Ruzzolini et al. [66]. Their study indicated that a diet enriched with oleuropein mitigated the pro-inflammatory tendencies of peritoneal macrophages in tumor-bearing rats.

Pro-inflammatory cytokines, such as IL-1β, TNF-α, and IL-6, were quantified in the serum due to their crucial roles in initiating and sustaining inflammatory responses, particularly in respiratory disorders. Our study revealed a significant increase in the levels of these cytokines, indicating an increase in the production of pro-inflammatory mediators as a result of exposure to noise and toluene. In contrast, rats supplemented with *Olea europaea* leaf extract maintained normal cytokine levels, suggesting its potential to mitigate the inflammation caused by these factors. 

IL-1β plays a crucial role in tissue destruction and edema [67]. In vitro studies have demonstrated that oleuropein influences both the release of IL-1β and its associated inflammatory actions. The oleuropein-rich leaf extract exhibited a time-dependent reduction in IL-1β expression in LPS-stimulated RAW264.7 cells [68]. Furthermore, in macrophages exposed to LPS, pre-treatment with oleuropein resulted in a decrease in IL-1β at both mRNA and protein levels by inhibiting the phosphorylation of IκB-α and the nuclear translocation of NF-κB [69]. Similarly, oleuropein countered IL-1β-induced inflammation by attenuating NF-κB and MAPK signaling [69].

IL-6 is another critical pro-inflammatory cytokine that plays a significant role in the immune response and inflammation. Elevated IL-6 levels have been associated with various inflammatory diseases, including respiratory disorders. Studies have shown that oleuropein can effectively reduce IL-6 levels. For instance, oleuropein treatment in LPS-stimulated macrophages led to a notable decrease in IL-6 production by inhibiting the TLR and MAPK signaling pathways, indicating its broad anti-inflammatory properties [70]. This suggests that oleuropein’s anti-inflammatory properties extend to the modulation of IL-6, further supporting its therapeutic potential.

TNF-α serves as a master regulator in orchestrating inflammation, deploying a range of mechanisms that intricately influence the inflammatory response. One notable aspect is its ability to stimulate the release of bioactive lipids, initiating vasodilation and intricately regulating essential adhesion molecules involved in guiding leukocytes to inflammatory sites [71].

The multifunctional inflammatory cytokine TNF-α, which is known for its capacity to stimulate the IL-1β and IL-6 release [72], emerges as a pivotal player in the pathogenesis of respiratory disorders within lung injury models. The intricate interplay of IL-1, IL-6, TGF-β, TNF-α, and ROS mediate deregulated inflammation.

**Figure 8:**
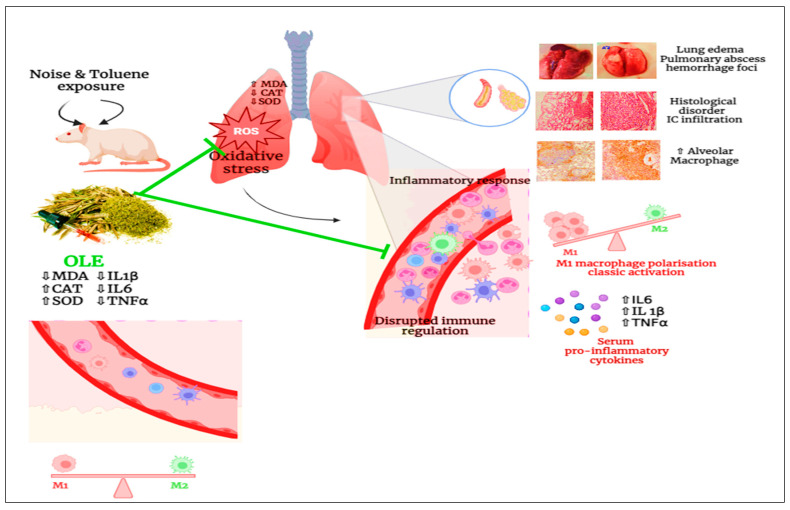
Schematic diagram illustrating the impact of pulmonary inflammation induced by chronic noise and toluene exposure.

Our study, summarized in Figure 8, hypothesizes that exposure to noise and toluene polarizes macrophages towards a pro-inflammatory type 1 subtype, thereby potentially increasing IL-1β, TNF-α, and IL-6 production via activation of the NF-κB signaling pathway. The administration of *Olea europaea* leaf extract is a promising strategy to alleviate the inflammation induced by toluene and noise exposure. 

Chronic exposure to noise and toluene harms the body, particularly the lungs, by inducing oxidative stress. This stress leads to an increased level of MDA, which disrupts the activities of catalase and SOD. Additionally, it causes disarray in the architecture of the pulmonary tissues. Co-exposure to noise and toluene increases pulmonary macrophage levels with classical activation, thus triggering an inflammatory response characterized by elevated levels of pro-inflammatory mediators such as TNF-α, IL-6, and IL-1β. This response results in vasodilation and increased vascular permeability, thereby promoting the infiltration of immune cells into pulmonary tissues. However, the administration of OLE reduced oxidative stress by decreasing MDA levels and enhancing antioxidant capacity. Concurrently, it also reduces the number of pulmonary macrophages, leading to a decrease in pro-inflammatory mediators and modulation of the inflammatory response. Moreover, OLE improves the architecture of pulmonary tissues.

## 5. Conclusions

In conclusion, the present study’s findings indicate that exposure to toluene, either alone or in combination with noise, significantly induces oxidative stress, inflammation, and histopathological alterations in the lungs. In contrast, noise exposure alone did not significantly change these parameters. Nevertheless, noise exposure increased the levels of pro-inflammatory cytokines, highlighting its role in immune response modulation. Notably, the administration of *Olea europaea* leaf extract demonstrated a protective effect, mitigating the adverse effects of toluene and noise. This study underscores the potential of natural compounds as protective agents against occupational hazards, suggesting their incorporation into health and safety protocols.

Future research should focus on elucidating the precise molecular mechanisms by which toluene disrupts the oxidative balance and induces inflammation, mainly through oxidative stress pathways and pro-inflammatory signaling cascades. Further investigation is warranted to understand how *Olea europaea* leaf extract modulates these pathways to restore homeostasis.

## Figures and Tables

**Table 1:** Antioxidant activities of *Olea europaea* L. leaf extract.

Contents	*Olea europaea* L. Leaf Extract
IC_50_ DPPH (µg/mL)	13.5 ± 0.75
Reducing power (µg/mL)	73.4 ± 4.08
Chelating capacity (µg/mL)	25.53 ± 2.58

**Table 2:** Percent inhibition of in vitro anti-inflammatory activity by different *Olea europaea* L. leaf extract concentrations using the bovine serum albumin denaturation method.

Concentration (µg/mL)	% OLE Denaturation	% Inhibition of BSA Denaturation by OLE	% DCF Denaturation	% Inhibition of Denaturation by DCF
10	78.82 ± 0.021	21.18 ± 0.06	93.11 ± 0.25	6.89 ± 0.14
50	51.76 ± 0.12	48.24 ± 0.25	74.45 ± 0.42	25.55 ± 0.21
75	38.66 ± 0.05	61.34 ± 0.52	58.82 ± 0.07	41.18 ± 0.35
100	29.92 ± 0.32	70.08 ± 0.025	44.71 ± 0.19	55.29 ± 0.71
150	13.78 ± 0.06	86.22 ± 0.16	27.06 ± 0.23	72.94 ± 0.02
200	4.20 ± 0.09	95.80 ± 0.1	21.01 ± 0.62	78.99 ± 0.54
250	2.69 ± 0.25	97.31 ± 0.03	12.77 ± 0.52	87.23 ± 0.63
400	1.68 ± 0.07	98.32 ± 0.41	2.02 ± 0.47	97.98 ± 0.01

**Table 3:** Percentage inhibition of in vitro anti-inflammatory activity by varied *Olea europaea* L. leaf extract concentrations using the egg albumin denaturation method.

Concentration (µg/mL)	% OLE Denaturation	% Inhibition of Albumin Denaturation by OLE	% DCF Denaturation	% Inhibition of Denaturation by DCF
10	94.20 ± 0.03	33.32 ± 0.25	66.68 ± 0.66	5.80 ± 0.36
50	65.67 ± 0.25	70.35 ± 0.36	29.65 ± 0.17	34.33 ± 0.70
75	35.33 ± 0.14	82.59 ± 0.27	17.41 ± 0.29	64.67 ± 0.27
100	22.03 ± 0.36	93.88 ± 0.80	6.12 ± 0.09	77097 ± 0.17
150	10.10 ± 0.61	99.21 ± 0.09	0.79 ± 0.77	89.90 ± 0.45
200	0.27 ± 0.48	99.61 ± 0.67	0.39 ± 0.07	99.73 ± 0.30

## Data Availability

All data and materials are available upon request.
